# Surgery and Psychiatry at Highest Risk: Physician Suicide Mortality by Specialty (2017–2022)

**DOI:** 10.1192/j.eurpsy.2026.10686

**Published:** 2026-07-31

**Authors:** C.-F. Sun, K. Irish, E. McCauley

**Affiliations:** 1 Department of Psychiatry, University of Washington School of Medicine; 2 Department of Statistics, University of Washington, Seattle, United States

## Abstract

**Introduction:**

Physician suicide is a critical public health issue, yet no original study in over a decade has evaluated mortality stratified by specialty. The COVID-19 pandemic may have altered the patterns.

**Table 1.** Table 1. Long description.

Specialty	Suicide Rate per 100,100/year
Surgery (total)	19.3
Psychiatry	15.9
Ophthalmology	9.5
Dermatology	7.5
Cardiology	7.2
Internal Medicine (total)	4.4

Note. Specialties are harmonized categories; “total” indicates aggregated specialty groups.

**Objectives:**

To understand the physician suicide trend in the US with a focus on differences across medical specialties and changes before versus after the COVID-19 pandemic.

**Methods:**

We analyzed U.S. physician suicides (2017–2022) using the National Violent Death Reporting System. Specialties were harmonized and linked to workforce denominators from the Area Health Resources File. We then calculated annual suicide rates per 100,000 physicians. Between-specialty incidence rate ratios (IRRs) were estimated relative to family medicine. Because workforce denominators differed between pre- and post-pandemic periods, within-specialty changes were assessed using IRRs comparing pre-pandemic (2017–2019) and post-pandemic (2020–2022). Sensitivity analyses examined the impact of missing specialty data.

**Results:**

We identified 724 physician suicides. Surgery had the highest mortality rate (19.3/100k/year; IRR = 7.96, 95% CI 4.4–14.4), followed by psychiatry (15.9/100k/year; IRR = 6.56, CI 3.6–12.1). We noticed suggestive elevations in ophthalmology (9.49/100k/year; IRR = 3.91, CI 1.78–8.61), dermatology (7.53/100k/year; IRR = 3.10, CI 1.91–8.07), and cardiology (7.21/100k/year; IRR = 2.98, CI 1.32–6.69), although confidence intervals were wide. (Table 1.) No specialties showed statistically significant changes in post-pandemic. Specialty was missing in 28% of cases, with the proportion increasing significantly from 25.8% to 30.7% post-pandemic (p = 0.036).

**Conclusions:**

Suicide mortality risk varies substantially across specialties, with surgery and psychiatry at highest risk. Post-pandemic increases in surgery and internal medicine highlight potentially vulnerable groups, while rising missingness in specialty reporting underscores the urgent need to improve surveillance of physician suicide.

**Disclosure of Interest:**

None Declared

## Long descriptions

### Table 1. Long description

The table consists of two columns: Specialty and Suicide Rate per 100,100/year. The data is presented in descending order of the suicide rate:

* Surgery (total): 19.3

* Psychiatry: 15.9

* Ophthalmology: 9.5

* Dermatology: 7.5

* Cardiology: 7.2

* Internal Medicine (total): 4.4

A note at the bottom specifies that specialties are harmonized categories and that total indicates aggregated specialty groups.

Navigate back to Table 1..

